# Elements of Natural Philosophy; Being an Experimental Introduction to the Study of the Physical Sciences

**Published:** 1840-04

**Authors:** 


Art. I.—*Elements of Natural Philosophy; being an Experimental Introduction to the Study of the Physical Sciences.* By Golding Bird, m.d., f.l.s., f.g.s., &c.—*London*, 1839. Small 8vo, pp. 407.

We quite agree with the author of this volume in the reason which suggested its compilation,—"the absence of any system of physics, sufficiently extended to include all those subjects with which men of education, especially members of a liberal and important profession like that of medicine, ought and are required to be familiar with; and at the same time not too diffuse to disgust or weary the student." The insulated treatises in Lardner's Cyclopædia, and the Library of Useful Knowledge, however valuable in themselves, are not adapted to convey to the student a *connected* idea of the objects of his pursuit; and they enter into details, in which it is not often desirable that one whose chief attention ought to be given elsewhere should follow them. The admirable Elements of Physics of Dr. Arnott have acquired a high and deserved popularity; but there are several objections to their becoming a text-book for the medical student. The first is a very serious one,—their price; another, even more grave, is the unfinished state in which they appear likely to remain. Moreover, we think that, for those whom it is an object to train in the school of physical science for the better employment of their faculties in the study and practice of medicine, something of a less diffuse manner and less copious illustration may be used with propriety, than that which Dr. Arnott has so judiciously employed as a means of attraction to the general reader.

If a general knowledge of physical science be made compulsory, as we trust it will be ere long, upon the medical student, we are disposed to believe that, if left to his own choice, he will seek to obtain it by attendance on lectures; provided these be made sufficiently attractive, as they may easily be, by experimental and other illustrations. The principles and general facts he will seek to impress more strongly upon his mind by reference to books; and he will require for this purpose a work containing as many of these as can be clearly stated, and made intelligible by application, within a small compass. This appears to have been the chief object of Dr. Golding Bird; who speaks of his work as "chiefly intended as a text-book for the student whilst attending lectures on physics." We are disposed to think very favorably of the mode in which he has fulfilled this purpose. A very large amount of important matter is comprised within a small compass, and it is almost uniformly expressed in a clear and simple manner. In this respect, we know of no other work at all to be compared to it. The newest doctrines in the rapidly advancing sciences of light, electricity, and electro-magnetism, are stated in a form which will render them easily comprehended by the student who has already made himself acquainted with the old; whilst he who is entering upon them uninformed is not perplexed by prolix discussions, but is led at once to what seems established as truth.

As an introductory work for the solitary student, however, we do not think it so well adapted. It is impossible to fulfil both these objects satisfactorily. The compression required in a text-book for lectures rarely suits the general reader. Dr. Alison's philosophic Outlines of Physiology are distasteful to the student, until he hears them expounded and illustrated by their author. As a mere *elementary* work, therefore, we do not recommend Dr. G. Bird's volume; but as an accurate and faithful guide to the study of physical science, we have great pleasure in strongly urging its merits upon our readers.

The scope of this work very nearly coincides with the first portion of the outline which we have given, in another part of this Number (Art. VI.), of a proper course of instruction in physical science. The following is a sketch of its contents. After an introductory discourse, in which the *forms* of matter are treated of, as well as the general principles of natural philosophy, the first nine chapters are devoted to the *Physics of Ponderable Matter*, under the following heads: I. General Properties of Atoms and Masses of Matter. II. Attractive Forces exerted between Masses. III. Bodies in Motion or General Dynamics. IV. Effects of Gravitation. V. Theoretical Action of the Simple Machines. VI. Fluids at Rest, or Hydrostatics. VII. Gases at Rest, Aerostatics, or Pneumostatics. VIII. Fluids in Motion, or Hydro- and Pneumo-dynamics. IX. Sonorous Vibrations of Ponderable Bodies, or Acoustics.

We think that a chapter on astronomy ought to have been given after the fourth. In this, without prolix details respecting the planetary system, the complex effects of the great principle of gravitation might have been displayed, as has been admirably done by Mrs. Somerville, in her "Connexion of the Sciences." We hold such *deduction* to be fully as valuable an exercise of the mind as *induction*; and, where a principle is known that accounts for *all* the phenomena to which it can be supposed to relate, its full application should be indicated. In no other respect have we any fault to find with this portion of the volume; and we trust its author will see the propriety of attending to our suggestion when the opportunity is afforded him.

The second division of the volume comprehends the *Physics of Imponderable Matter*,—a term which we are disposed to think objectionable both in itself and as not expressing the present state of opinion as to the nature of electricity, magnetism, &c. This, however, is not a matter of great importance, since we do not perceive that the text has been affected by the idea contained in the title. This division contains sixteen chapters, of which the titles are as follows: X. Magnetism. XI. Primary Phenomena of Ordinary Electricity. XII. and XIII. Consequences of Electrical Induction. XIV. Phenomena of Atmospheric Electricity. XV. Voltaic Electricity. XVI. Electro-Dynamics. XVII. Electro-Dynamic Induction. XVIII. Thermo-Electricity. XIX. Organic

Electricity. XX. General Properties and Catoptric Phenomena of Unpolarized Light. XXI. Dioptric Phenomena of Unpolarized Light. XXII. Chromatic Phenomena of Unpolarized Light. XXIII. Phenomena of Double Refraction and Rectilinear Polarization. XXIV. Chromatic Phenomena of Polarized Light. XXV. Optical Apparatus, and the Eye considered as an Optical Instrument.

As electricity is Dr. Golding Bird's favorite object of pursuit, and a department of science in which he has greatly distinguished himself, it is not to be wondered at that rather a disproportionate space should be allotted to it in his work. We speak, however, more with regard to the *objects* of his volume than as to the relative importance of the *principles* of gravitation and electricity in an abstract point of view. It does appear to us, however, that the details given under the latter head might be sufficiently abbreviated to admit of the corresponding amplification which we have suggested in the former.

We are at a loss to account for the omission of *heat* from this division of the work. It unquestionably stands in very close relation both to light and electricity; and, if forms of imponderable matter can be conceived of, it is certainly entitled to rank among them. No less than one fourth of the whole volume is devoted to light; and here, too, we think that a little retrenchment may be advantageously made, for the admission of a subject so nearly allied, and of so much importance.

The volume, as it at present stands, approaches so closely to our idea of what such a treatise *should be*, that we have dwelt longer upon these trifling defects than we otherwise should have thought it worth while to do. The student who had made himself master of it, amended as we had proposed, would then be qualified to enter with advantage upon the study of chemistry. This would lead him to mineralogy on one side, and physiology on the other; and these two branches of science meet again in geology. We think that an additional volume, embodying the principles of chemistry (and especially dwelling upon the inorganic division of this science) with those of mineralogy and dynamical geology, would form a most appropriate continuation of the present treatise; and it could scarcely be executed by any one better qualified for the task than the author of this work.

